# Semaglutide in the Real World: Attitudes of the Population

**DOI:** 10.3390/pharmacy13050128

**Published:** 2025-09-04

**Authors:** Doris Rušić, Toni Durdov, Ivona Jadrijević, Ana Šešelja Perišin, Dario Leskur, Joško Božić, Mila Marie Klusmeier, Josipa Bukić

**Affiliations:** 1 Department of Pharmacy, University of Split School of Medicine, Šoltanska 2A, 21000 Split, Croatia; drusic@mefst.hr (D.R.); toni.durdov@mefst.hr (T.D.); ivona.jadrijevic@mefst.hr (I.J.); milamarie.klusmeier@dz-sdz.hr (M.M.K.); jbukic@mefst.hr (J.B.); 2 Department of Pathophysiology, University of Split School of Medicine, Šoltanska 2A, 21000 Split, Croatia; jbozic@mefst.hr; 3 Department of Laboratory Medicine and Pharmacy, Faculty of Medicine, Josip Juraj Strossmayer University of Osijek, Josipa Huttlera 4, 31000 Osijek, Croatia

**Keywords:** semaglutide, questionnaire, weight loss

## Abstract

Background: Clinical experience with semaglutide in patients with type 2 diabetes mellitus shows that its benefits extend far beyond glucose regulation. This study examines whether this drug is indeed popular among the Croatian population and explores whether factors such as gender or proximity to the healthcare sector influence its potential use, attitudes toward weight loss, and knowledge regarding its application and possible adverse effects. Methods: This was a cross-sectional population study. In this study we focused on the brand name Ozempic^®^ for semaglutide as it is the most commonly searched term for semaglutide. Results: The study included 290 participants, most of who were women (*N* = 243, 83.8%). As many as 214 (73.8%) people stated they had heard of Ozempic^®^; however, there was no significant difference in whether people had heard of Ozempic^®^ if they had type 2 diabetes mellitus (*p* = 0.415). In total, 23.4% of people stated they knew someone who took Ozempic^®^. Women were significantly more likely to feel pressure about their appearance than men, with 51.1% of men reporting no pressure at all compared to only 39.9% of women (*p* = 0.015). A majority of participants agreed that social media strongly affects perception on the use of medications for weight loss (73.8%). Individuals with a family member in the healthcare field were significantly more informed about the possible adverse reactions of semaglutide compared to those without such a connection. Among participants without a healthcare professional in the family, 75.0% reported being unaware of potential adverse effects, compared to 47.9% of those with a family member in healthcare. Moreover, participants with a healthcare professional in the family were more likely to know the correct route of administration for Ozempic^®^ (68.1% vs. 54.6%, *p* = 0.025); Conclusions: The results of this study show that three-quarters of people had heard of Ozempic^®^, regardless of whether they had an indication for its use or not. In addition, the results indicate that although both men and women share satisfaction with their bodies, women feel more pressured by societal expectations related to their appearance.

## 1. Introduction

Clinical experience with semaglutide in patients with type 2 diabetes mellitus shows that its benefits extend far beyond glucose regulation [1]. In the SUSTAIN trial, patients with type 2 diabetes were administered a once-weekly subcutaneous injection of semaglutide and compared to patients receiving placebo for 30 weeks. A significant reduction in weight and HbA1c was observed in the semaglutide group. The most common adverse reactions were gastrointestinal in nature [2,3].

Unlike most other older medications for the treatment of type 2 diabetes mellitus, glucagon-like peptide-1 receptor agonists (GLP-1 RAs) promote weight loss addressing the common type 2 diabetes comorbidity, obesity. Among other actions, GLP-1 RAs reduce appetite by acting on the arcuate nucleus at the hypothalamic level, and increase satiety, leading to less food intake. Specifically, it reduces the preference for palatable foods, salty and sweet foods, and foods high in fat [1,3]. Among individuals with type 2 diabetes that were also at high risk of cardiovascular disease, semaglutide significantly decreased the occurrence of cardiovascular events compared to standard care [4,5]. Compared to dulaglutide, semaglutide proved to be superior for weight loss and glycemic control with a similar safety profile [6]. Furthermore, in patients with type 2 diabetes and chronic kidney disease who are at high risk of heart failure, semaglutide once weekly increased time to the first heart failure events or cardiovascular death [7]. The results of the STEP trial confirmed significant and sustained weight loss alongside improvement in cardiometabolic risk factors. The most common adverse reactions were gastrointestinal and mild to moderate. There was no need for treatment discontinuation [8,9]. The benefits of semaglutide in patients with obesity surpass simple weight loss. Compared to placebo, semaglutide achieved a 20% reduction in major adverse cardiovascular events and provided beneficial kidney outcomes in individuals with obesity [10].

The literature reports various rates for the discontinuation of semaglutide. A study on 175 patients using semaglutide for weight loss reported a discontinuation rate due to adverse effects of 4.1% [11]. Furthermore, the SELECT trial investigating the long-term weight loss effects of semaglutide in obesity reported a significantly greater rate of permanent discontinuation of semaglutide (16.6%) compared to an 8.2% rate of discontinuation in the placebo group [12]. A systematic review with meta-analysis on the efficacy and safety of semaglutide for weight loss in obese patients without diabetes showed a 6% rate of discontinuing semaglutide due to adverse effects. The risk of discontinuation was twice higher than in the placebo group [13].

The weight loss resulting from the introduction of semaglutide in the treatment of diabetes type 2 fueled the population’s interest in this drug and spiked consumer demand [14,15]. Overall, there is an increasing trend in online searches for semaglutide observed over time with a number of queries on how to obtain the drug [16]. A shift from searches for traditional weigh loss techniques to those for semaglutide has been observed [17]. It seems that social media pressure has reduced rates of obesity in the United States [18]. Analysis of 5310 adverse drug reactions showed that semaglutide was used off-label in 20.97% of cases and for unapproved indications in 17% of cases [19]. In fact, today semaglutide is approved for obesity under the brand name Wegovy^®^ as a once-weekly subcutaneous drug [3].

The global epidemic of obesity and its increasing rate puts a great economic burden on societies; medication-based treatment of obesity with drugs such as semaglutide may halve the costs, depending on the other comorbidities [18,20,21]. However, some studies came to the conclusion that the use of semaglutide just for weight loss is not cost-effective [22]. Nevertheless, it is encouraging that people will take medications for weight loss if supported by their insurance coverage [23]. However, a published analysis shows that among the approved anti-obesity drugs, semaglutide shows a continuously increasing trend in the annual reporting of serious adverse events, which may be a potential reason for caution [24].

In this study, we focused on the brand name Ozempic^®^ for semaglutide as it is the most commonly searched term for semaglutide [25]. We wanted to investigate where semaglutide stands in the real world. The study investigates if this drug is in fact popular among the Croatian population and whether factors such as sex or closeness to the healthcare sector affect the possible use of the drug, attitudes towards weight loss, and knowledge on the use of this drug and its possible adverse reactions.

## 2. Materials and Methods

This was a cross-sectional population study. The questionnaire was designed in the Croatian language by a pharmacist and a doctor of medicine and comprised 20 questions. The first part gathered data on semaglutide awareness using its registered brand name in Croatia, Ozempic^®^, in terms of whether participants had heard of this drug with a simple yes/no question. They were also asked if they knew its indications and to choose possible adverse reactions they related to the mentioned medication. Some of the questions asked the participants whether they used diets, medications or dietary supplements for weight loss, and to rate satisfaction with their body on a 5-point Likert scale. Participants were asked to grade the societal pressure they felt to look a certain way on a 5-point Likert scale, and how often they considered weight loss, also on a 5-point Likert scale. In its final part, the questionnaire gathered sociodemographic data of participants including sex, education, average income of household, and whether they had a family member in the healthcare sector. Prior to the main survey, the questionnaire was pilot tested on eight individuals from the target population to assess clarity, comprehensibility, and length. As part of the pilot, four participants completed the questionnaire a second time after a 7-day interval to allow a preliminary assessment of temporal stability. Responses showed no discrepancies in factual items and high agreement in Likert scale ratings, suggesting good short-term reproducibility. Minor wording adjustments were made based on participant feedback to improve clarity. The questionnaire used in the study is given as Supplementary File S1.

The link with the questionnaire was distributed in a pseudo-snowballing manner. The study authors distributed the link to their family and friends, asking them to distribute it further to their family and friends. All Croatian residents older than 18 years of age were eligible for participation in the study. The study started in September of 2024 and lasted for three months. The study was approved by the Ethics Committee of the University of Split School of Medicine. Filling out the questionnaire was deemed informed consent. Participants received no compensation for enrolling in the study and could cancel their participation at any time without repercussions.

Study participants were observed based on sex, women vs. men, and whether they had a healthcare professional in the family, yes vs. no. Results are presented as whole numbers and percentages. Prior to statistical analysis, participants’ answers to the question of how many hours a day they dedicate to their physical appearance were clustered into the following groups: ≤0.25 h, <0.25–0.5 h, <0.5–1 h, <1–2 h, ≥2 h. Chi-square tests and Fisher’s exact tests were performed where necessary to compare answers between groups. Statistical analysis was conducted using IBM SPSS Statistics software (version 25). Statistical significance was set at *p* < 0.05.

## 3. Results

This study included a total of 290 participants from Croatia. Most of the participants were women (*N* = 243, 83.8%) while there were only 47 men (16.2%). Participants were predominantly between 26 and 35 years old (*N* = 67, 23.1%); only 7.9% of people surveyed were older than 65 years (*N* = 23). More than half of study participants (*N* = 154, 53.1%) stated they had finished university graduate studies and roughly 20% stated their highest degree was from high school. One-third of our study participants stated they had a family member working in healthcare. Detailed characteristics of the study participants are given in Table 1.

In this study 214 (73.8%) people stated they had heard of Ozempic^®^. There was no significant difference in whether people had heard of Ozempic^®^ based on whether they had type 2 diabetes mellitus (*p* = 0.415) or a family member working in a healthcare field (*p* = 1.108).

**Figure 1:**
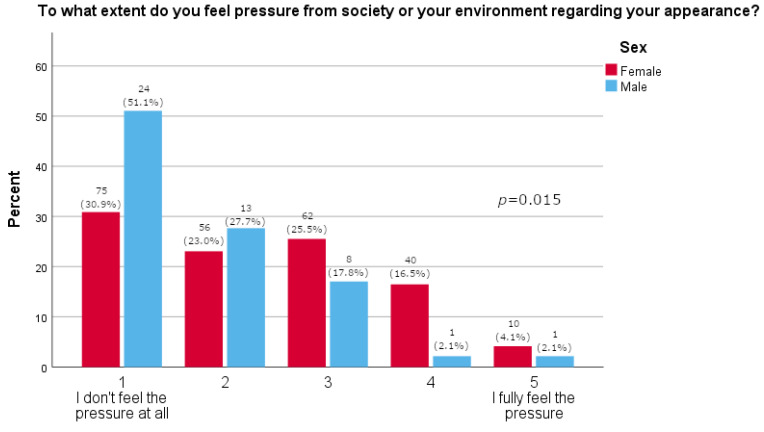
Pressure regarding appearance (5-point Likert answers are presented on x-axis; percentage of participants is given on y-axis).

**Figure 2:**
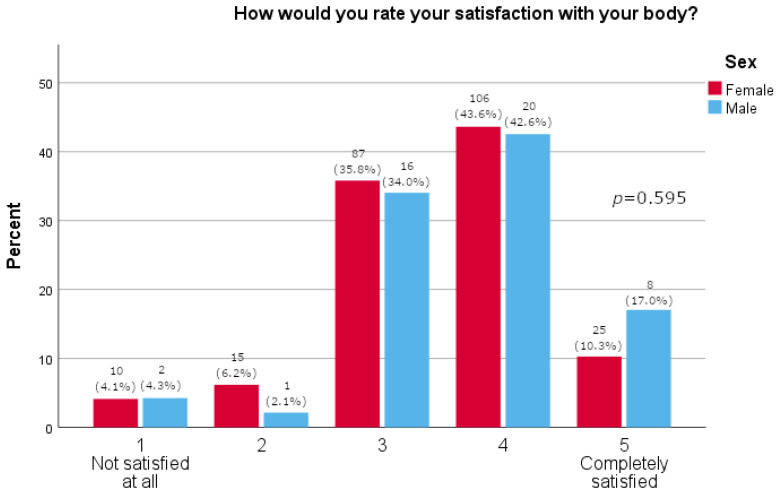
Body satisfaction (5-point Likert answers are presented on x-axis; percentage of participants is given on y-axis).

**Figure 3:**
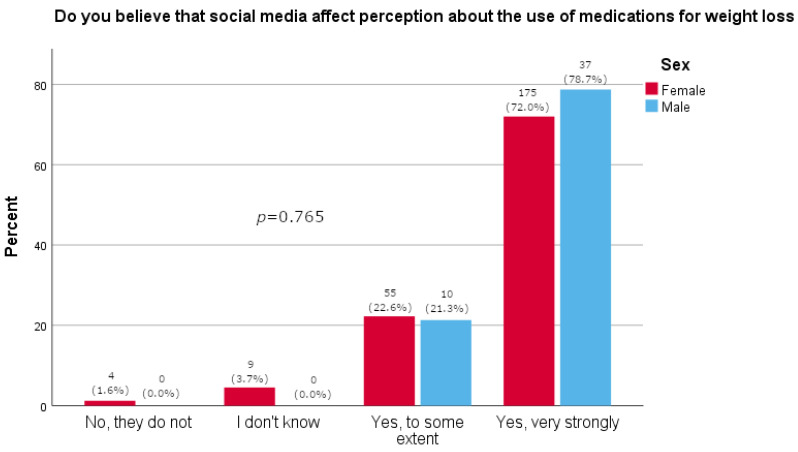
Participants’ opinions on the effect that social media has on weight loss medication (participants’ answers are presented on x-axis; percentage of participants is given on y-axis).

**Figure 4:**
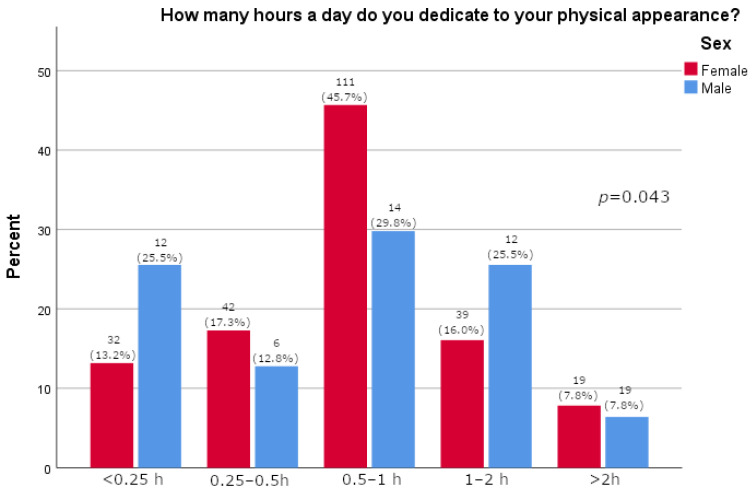
Time in day dedicated to physical appearance relative to sex (participants’ answers are presented on x-axis; percentage of participants is given on y-axis).

Women more often reported they often thought about losing weight (*p* = 0.0163), as shown in Table 2. Furthermore, women were significantly more likely than men to feel pressure about their appearance (*p* = 0.015, Figure 1). There was no significant difference between the sexes in satisfaction with one’s body (*p* = 0.595, Figure 2). A majority of participants agreed that social media strongly affected their perception of the use of medications for weight loss (73.1%, Figure 3). The results of this study showed that women tend to spend more time on their physical appearance compared to men, with around a quarter of men not dedicating more than 15 min a day to their physical appearance, while half of women dedicated between 30 min and 1 h (*p* = 0.043, Figure 4).

In this research, 155 (53.4%) people stated they would use Ozempic^®^ if recommended by their physician, 68 (23.4%) if they were unsatisfied with their body, 41 (14.1%) if it were recommended to them by a friend or someone they knew, and 16 (5.5%) under the influence of information from the internet. Only 6 (2%) people reported they or someone they knew got Ozempic^®^ via the internet and 9 (3%) that they got it in a pharmacy without a prescription, whereas for 49 (16.9%) it was prescribed when they pressured their physician. Other answers included semaglutide left over from a diabetic member of the household, one person stated a secret connection, another had a diabetologist friend, and one was uncertain about how the person obtained semaglutide.

Significantly more people who had a family member working in a healthcare field knew the correct route of administration of Ozempic^®^ (*p* = 0.025) (Table 3). In total, 23.4% of people stated they knew someone who took Ozempic^®^.

People who had a family member in a healthcare field were significantly more knowledgeable about the possible adverse reactions of semaglutide compared to others. Overall, most people in the study identified nausea and gastrointestinal disturbances as possible adverse reactions of semaglutide (26.2% and 23.8%). Acute pancreatitis was recognized by 16.9%, while anaphylactic reaction, gastritis, increased heart rate, and cholelithiasis were recognized by less than 10% of participants. Thyroid cancer was identified as a possible adverse reaction by just 5.9% of participants (Table 4).

## 4. Discussion

As many as 73.8% of study participants reported that they had heard of Ozempic^®^. This finding was independent of whether they had type 2 diabetes mellitus. This is an interesting finding, as it is not expected that a person would hear of a prescription drug if they do not have an indication for it. Our results may be skewed since a majority of the participants were younger persons and women in great proportion. These findings, however, are in line with a recent study conducted among youth (14–24 years) in which 73.6% stated they had heard of drugs such as Ozempic^®^ and Wegovy^®^ [27]. Our study included 290 participants, most of who were women (*N* = 243; 83.8%); furthermore, most participants were between 26 and 35 years of age (*N* = 67; 23.1%). This is particularly important as women are generally expected to have a greater inclination toward using weight-loss medications, and younger individuals are more likely to spend time on social media and obtain news and information from these platforms compared to older adults [27]. In fact, the study’s results show that women reported thinking about weight loss significantly more often than men (*p* = 0.016) and experiencing significantly greater pressure related to their appearance (*p* = 0.015). Interestingly, there was no difference observed between the two sexes in reported self-satisfaction with one’s body (*p* = 0.595). The results were further supported by the fact that significantly more men reported not using anything (diets, supplements, drugs) for weight loss (72.3% vs. 50.2%, *p* = 0.005). A Danish study confirmed that women more often sought and took semaglutide for weight loss [28]. By comparison, the literature reports that young men have a more negative and hesitant perception of the use of medications for weight loss and prefer healthy life-style behaviors [27]. In our study, as few as four people (1.6%), all women, reported they would use medications for weight loss. Furthermore, analyses show that women are more prone to adverse drug reactions related to semaglutide, likely due to social pressure leading them to use the drug more frequently than men, confirming that they should be the primary focus of this and similar studies, although some studies indicate that semaglutide is more effective in reducing weight in women than men [19,29].

According to the results of our study and routes by which people obtain Ozempic^®^, we could estimate that 16.9% of people taking Ozempic^®^ were reimbursed for the medication and 5% were paying out of pocket for the medication. There is an obvious discrepancy, and it is likely that even more people would consume it if it were available to them under reimbursement. Regulatory authorities lack effective means to control how the drug is portrayed on social media, creating another significant health risk for patients [30]. For example, a study looking at tweets related to semaglutide found that almost 93% did not mention any adverse reactions, while 63% discussed positive efficacy. However, more than 74% included a link to scientific data [31]. Fortunately, only around 5% of our study participants reported they would take Ozempic^®^ under the influence of information from the internet, while more than half would only take it on a physician’s recommendation. The literature documents that young people using social media are aware that social media contributes to the glamorization of using medications for weight loss, promotes ideals of thinness and weight stigma, contributes to misconceptions about medications, and encourages the unnecessary use of certain medications for weight loss, ultimately leading to drug shortages [27]. As many as 73.8% of our study participants reported believing that social media strongly influences people’s perception of the use of weight loss medications.

In this study, 66.2% of participants stated they were not informed about possible adverse reactions to semaglutide. There were significantly fewer people who stated they were not informed about the adverse reactions in the group that stated they had a family member who was a healthcare worker, 47.9% vs. 75.0%, *p* < 0.001. If we take a look at the Summary of Product Characteristics for Ozempic^®^ in European Medicines Agency or Croatian Agency for Medicinal Products and Medical Devices website, we find that diarrhea and nausea occur quite frequently, more precisely in more than one person in ten. Furthermore, we find that it has been documented that other gastrointestinal disturbances, as well as gastritis and cholelithiasis, commonly occur in more than 1% and less than 10% of those taking the medication. Increased heart rate and acute pancreatitis are documented as uncommon adverse reactions occurring in fewer than one person out of a hundred. Finally, anaphylactic reactions are listed as rare, but still recognized as a possible adverse reaction to this medication occurring in less than one person in ten thousand. Preclinical safety data list non-lethal thyroid C-cell tumors in rodents as related to a mechanism to which rodents are particularly sensitive. The relevance to humans is considered to be low; however, it cannot be completely excluded [26]. Notably, most people in this study identified nausea and gastrointestinal disturbances as possible adverse reactions to semaglutide (26.2% and 23.8%), which are also established to be the most frequent adverse reactions to the drug [1,32]. Acute pancreatitis was recognized by 16.9%, while anaphylactic reaction, gastritis, increased heart rate, and cholelithiasis were recognized by less than 10% of participants. Thyroid cancer was observed as a possible adverse reaction by just 5.9% of participants. It should be noted that as the population taking a certain medication grows, so do the safety risks and concerns [33]. Recently, there have been analyses of safety signals potentially linking semaglutide with optic nerve and retinal adverse events as well as certain neuropsychiatric adverse events [34,35,36,37,38]. In this study, knowledge of adverse reactions was more frequently observed in the group of participants who had a family member who was a healthcare worker, indicating that these people are better informed on the safety of administration of the drug and its possible adverse reactions. It would have been interesting to explore sources of information for our study participants and cross-reference them with these results. A study that explored the quality of information about semaglutide on TikTok found that 71% of videos did not mention any risks related to semaglutide, while just 11.9% mentioned one or more serious risks [30]. Another study on 189 YouTube videos with a total view count of over 45 million showed that YouTube-verified health source channels had a higher average view count than others, but this was not significant. Furthermore, short videos lacked information on the prevalence of adverse reactions [39].

The results of this study further confirm that the off-label use of semaglutide for cosmetic purposes, specifically weight loss, has surged in popularity [14]. Published data report that 46.3% of the public supports physician discretion for off-label prescribing, but 58% are somewhat concerned about supply shortages with 63% expressing concerns related to safety in the context of off-label use [40]. According to the results of our study, there were only 11 (3.8%) people that had diabetes. However, as many as 73.8% stated they had heard of Ozempic^®^, and there was no significant difference based on whether they had a family member who was a healthcare worker (*p* = 0.109). A little less than a quarter (23.1%) of our participants stated that they knew someone who took Ozempic^®^ and there was no significant difference based on the presence of a healthcare worker in the family. One might expect that people with a healthcare worker in the family might have greater access to prescription drugs such as semaglutide and hence know more people using it, but this was not the case in our study, indicating that a number of people obtain prescription drugs by bypassing the regulations. For comparison, in a recent study, 26.1% of people stated they knew someone using Ozempic^®^ or Wegovy^®^, with women reporting it significantly more often (*p* = 0.020) [27]. Among our participants, 16.9% stated they or someone they knew demanded their physicians prescribe them semaglutide, 3.4% bought it in a pharmacy without a prescription, and 2% obtained it from the internet. Other means included semaglutide being left over from a diabetic member of household, a secret connection, having a friend diabetologist, and being uncertain how the person obtained semaglutide. Interestingly, a study conducted among 127 physicians prescribing subcutaneous semaglutide for weight loss found that two-thirds of them required lifestyle changes prior to treatment initiation, but only 60% reported they would not prescribe the medication in persons with eating disorders. Only 23% considered physical activity as essential for achieving weight loss, while 48% deemed it essential for weight loss maintenance [41]. Our study highlights the challenging position physicians and pharmacists face when pressured by patients or acquaintances to prescribe drugs on demand and reveals alternative channels through which semaglutide is dispensed in Croatia. The results of this study also indicate that physicians face greater pressure compared to pharmacists. The regulation on internet sales of medicinal products in Croatia at the time of writing this manuscript is not yet in force, and the draft permits internet sales options only for over-the-counter medicinal products, while semaglutide in all its forms is marketed as a prescription-only medicinal product. As many people that are interested in using semaglutide do not have an indication for semaglutide, they tend to turn to alternative routes. These practices may expose them to even greater health risks as they may face counterfeit medicines from unregulated supply chains. A study investigating the safety of semaglutide products sold online without a prescription documented a significant presence of illegal online pharmacies that promote off-label use of semaglutide, which attract millions of visits monthly. The study established that all obtained vials were probably substandard and falsified products, and found discrepancies in labelled amounts of semaglutide [14].

Semaglutide, under the brand name Ozempic^®^, is formulated in vials for subcutaneous application and should not be used as an intramuscular or intravenous injection [26]. In this study, significantly more people who had a healthcare worker in their family knew the correct route of administration of Ozempic^®^ (68.8% vs. 54.9%, *p* = 0.022). Per os was chosen by 35.4% of participants, 30.1% in the group that had a family member in healthcare and 37.9% among the other participants. This result is not surprising, as semaglutide as an active moiety is marketed in a per os once-daily formulation for the treatment of type 2 diabetes mellitus, although under a different brand name [42,43]. It is expected that the new formulation will reduce the burden associated with injections and allow for greater patient compliance. A number of other oral semaglutide formulations are in different stages of clinical development [4].

This study has a number of limitations that need to be taken into consideration. The population that participated in the study is skewed towards the female sex. This comes as no surprise, as the study showed that women feel more pressure about their appearance then men, and it is possible they were already knowledgeable on the topic and more eager to participate than their male counterparts. However, one might expect that women are more likely to use drugs, diets and supplements for weight loss, therefore in fact being the target population of this and similar research. This was also confirmed with the results of this study, as women reported dieting for weight loss significantly more often compared to men (42.4% vs. 14.9%, *p* < 0.001). Interestingly, no significant difference was observed between the two sexes for the use of dietary supplements or drugs for weight loss (*p* = 0.574; *p* = 0.377). One can also argue that men who use drugs for weight loss are less likely to report it. It is also possible that the higher proportion of female participants in our sample led to an inflated estimate of the percentage of people who had heard of semaglutide. For example, a 2024 study with approximately 65% female respondents found that 47% considered semaglutide a potential weight-loss medication. In contrast, a 2023 study of the general population, with about 62% women, reported that only 33% recognized semaglutide as a weight-loss medication. In our study, however, this percentage was nearly 77% [44,45]. On the other hand, a 2024 study investigating the relationship of demographic factors to patient perspectives on incretin-based weight loss medications did not find significant differences in awareness or use of these medications between the two sexes [46]. The year the mentioned studies were conducted might also be relevant for the matter. Furthermore, the sample size is rather small, but this study showed interesting and somewhat expected results. The distribution of the questionnaire and the fact that it was only available in digital form favored a younger population, making the questioned population more likely to be influenced by social media. Also, the pseudo-snowballing method of distribution may have favored the authors’ family and friends. This sampling method may have led to bias in the sample, resulting in more people that had a healthcare professional in the family compared to the general population, explaining the relatively high proportion of respondents who had a family member in healthcare (32.4%). This may affect the generalizability of our results, and does not allow for response rate calculation. Due to the nature of the pseudo-snowball sampling method used, the total number of individuals who received the survey invitation is not known, and therefore an accurate response rate could not be calculated. A limitation of this study is that the questionnaire underwent only limited reliability assessment during the pilot phase, without full psychometric validation; however, given its focus on factual awareness and simple subjective ratings, this approach was considered sufficient for the study’s descriptive objectives.

## 5. Conclusions

The results of this study demonstrate that approximately three-quarters of participants had heard of semaglutide, regardless if they had an indication to use it or not. Participants with a healthcare professional in their family possessed greater knowledge of the potential adverse reactions associated with semaglutide compared to those without such a connection. The findings of this study also suggest that social media exerts a substantial influence on health-related behaviors, underscoring the need for stricter regulation of “drug marketing” on these platforms. Furthermore, the results indicate that, although men and women report similar levels of body satisfaction, women experience greater societal pressure regarding their appearance. This heightened pressure may render women disproportionately vulnerable to adverse drug reactions and increase their susceptibility to related conditions, including mental health disorders.

## Figures and Tables

**Table 1:** Study participants’ characteristics.

Characteristic	*N* (%)
Total	290 (100.0%)
Sex	
Women	243 (83.8%)
Men	47 (16.2%)
Age (years)	
18–25	53 (18.3%)
26–35	67 (23.1%)
36–45	42 (14.5%)
46–55	56 (19.3%)
56–65	49 (16.9%)
65+	23 (7.9%)
Highest level of education achieved	
Postgraduate scientific master’s studies or postgraduate university (doctoral) studies	19 (6.6%)
University graduate studies; specialist graduate professional studies; postgraduate specialist studies	154 (53.1%)
University undergraduate studies; professional undergraduate studies	44 (15.2%)
General secondary education (gymnasium); four-year and five-year vocational secondary education	63 (21.7%)
Three-year vocational education	7 (2.4%)
Primary education or less	3 (1.0%)
Family member working in healthcare	
Yes	94 (32.4%)
No	196 (67.6%)
Has type 2 diabetes mellitus	
Yes	11 (3.8%)
No	279 (96.2%)

**Table 2:** Sex differences in answers to questionnaire.

	Men (*N* = 47)	Women (*N* = 243)	*p*-Value	Total (*N* = 290)
How often do you think about losing weight?				
Very often	5 (10.6%)	28 (11.5%)	0.016	33 (11.4%)
Often	5 (10.6%)	69 (28.4%)		74 (25.5%)
Sometimes	22 (46.8%)	107 (44.0%)		129 (44.5%)
Never	15 (31.8%)	39 (16.0%)		54 (18.6%)
Do you know that Ozempic^®^ is used for weight loss, although it was developed for the treatment of type 2 diabetes mellitus?				
Yes	35 (74.5%)	188 (77.4%)	0.667	223 (76.9%)
No	12 (25.5%)	55 (22.6%)		67 (23.1%)
Which of the following have you used for weight loss?				
Diets	7 (14.9%)	103 (42.4%)	<0.001	110 (37.9%)
Dietary supplements	7 (14.9%)	29 (11.9%)	0.574	36 (12.4%)
Medications	0(0.0%)	4 (1.6%)	0.491	
Nothing	34 (72.3%)	122 (50.2%)	0.005	156 (53.8%)

**Table 3:** Answers to questionnaire relative to having a family member in the healthcare sector.

Family Member Health Care Worker	Yes (*N* = 94)	No (*N* = 196)	*p*-Value	Total (*N* = 290)
What is the route of administration of Ozempic^®^?				
Subcutaneous	64 (68.1%)	107 (54.6%)	0.025	171 (59.4%)
Intravenous	1 (1.1%)	14 (7.1%)		15 (5.2%)
Per os	28 (29.8%)	74 (37.8%)		102 (35.4%)
Did you or someone you know take Ozempic^®^ for weight loss?				
I took it and I know someone who took it	0(0.0%)	1 (0.5%)	0.407	1 (0.3%)
Someone I know took it	26 (27.7%)	41 (20.9%)		67 (23.1%)
No	68 (72.3%)	154 (78.6%)		222 (76.6%)

**Table 4:** Knowledge of adverse reactions relative to having a family member in the healthcare sector.

Family Member Health Care Worker/Adverse Drug Reaction	Frequency of Adverse Reaction According to SmPC for Ozempic^®^ [26]	Yes (*N* = 94)	No (*N* = 196)	*p*-Value	Total (*N* = 290)
I am not informed	-	45 (47.9%)	147 (75.0%)	<0.001	192 (66.2%)
Nausea	≥1/10	40 (42.5%)	36 (18.4%)	<0.001	76 (26.2%)
Gastrointestinal disturbances	≥1/100 and <1/10	37 (39.4%)	32 (46.4%)	<0.001	69 (23.8%)
Acute pancreatitis	≥1/1000 and <1/100	21 (10.7%)	28 (29.8%)	<0.001	49 (16.6%)
Anaphylaxis	≥1/10 000 and <1/1000	13 (13.8%)	10 (5.1%)	0.010	23 (7.9%)
Gastritis	≥1/100 and <1/10	12 (12.8%)	10 (5.1%)	0.021	22 (7.6%)
Increased heart rate	≥1/1000 and <1/100	10 (10.6%)	9 (4.6%)	0.052	19 (6.6%)
Cholelithiasis	≥1/100 and <1/10	12 (12.8%)	6 (3.1%)	0.001	18 (6.2%)
Thyroid cancer	not reported	9 (9.6%)	8 (4.1%)	0.063	17 (5.9%)

SmPC—Summary of Product Characteristics.

## Data Availability

Raw data are available from the corresponding author upon reasonable request.

## Supplementary Materials

The following supporting information can be downloaded at: https://www.mdpi.com/article/10.3390/pharmacy13050128/s1, Questionnaire S1: Questionnaire Regarding the Use of Semaglutide in Croatia.
