# Supplementary material for: Semaglutide in the Real World: Attitudes of the Population
Source: Pharmacy (Basel). 2025 Sep 4;13(5):128. doi: 10.3390/pharmacy13050128 (PMC12452689; doi:10.3390/pharmacy13050128)
Supplement: Supplementary file 1 [file pharmacy-13-00128-s001.zip › pharmacy-3819490-supplementary.pdf]

Questionnaire Regarding the Use of Semaglutide in Croatia

1. Do you have diabetes?
   a) Yes
   b) No

2. Have you ever heard of Ozempic®?
   a) Yes
   b) No

3. How is Ozempic® administered?
   a) Orally
   b) Intravenously
   c) Subcutaneous injection

4. Are you aware that Ozempic® is used for weight loss, although it is primarily intended for diabetes treatment?
   a) Yes
   b) No

5. Have you or someone you know used Ozempic® for weight loss?
   a) I have used it
   b) I know someone who has used it
   c) None of the above

6. If you or someone you know used Ozempic®, how was it obtained?
   a) Prescribed by a doctor at my request
   b) Bought from a pharmacist without a prescription
   c) Obtained online
   d) Other: _______

7. What motivated (or would motivate) you to consider using Ozempic® for weight loss? (select all that apply)

a) Doctor’s recommendation
b) Recommendation from a friend/acquaintance
c) Information from the internet/social media
d) Dissatisfaction with body image
e) Other: _______

8. Which risks or side effects do you associate with the use of Ozempic®? (you may choose more than one)

a) Nausea
b) Digestive issues
c) Gastritis
d) Increased heart rate
e) Gallstones
f) Acute pancreatitis
g) Anaphylactic reaction
h) Increased risk of thyroid cancer
i) I am not informed
j) Other: _______

9. How satisfied are you with your body?

1 – Completely dissatisfied

2 – Dissatisfied

3 – Neither satisfied nor dissatisfied

4 – Satisfied

5 – Completely satisfied

10. How often do you think about losing weight?

a) Never
b) Sometimes
c) Often
d) Very often

11. Have you ever used other products or methods for weight loss?

a) Yes, medications
b) Yes, diets
c) Yes, dietary supplements
d) No

12. To what extent do you feel pressure from society or your surroundings about your appearance? (1 = not at all, 5 = completely)

1 - Not at all

2 - Slightly

3 - Moderately

4 - Very

5 – Completely

13. How important is information about side effects before deciding to use a drug or supplement? (1 = not important at all, 5 = very important)

1 - Not important at all

2 - Slightly important

3 - Moderately important

4 - Important

5 - Very important

14. Do you believe social media influences people’s perception of using medications for weight loss?

a) Yes, strongly influences
b) Yes, somewhat influences
c) No, does not influence
d) I don't know

15. How many hours per day do you spend on physical appearance?

____

16. Gender:

a) M

b) F

17. Your age group:

a) 18–25

b) 26–35

c) 36–45

d) 46–55

e) 56–65

f) 65+

18. Average monthly household income (in euros):

____

19. Highest level of education completed:

a) Primary school or less

b) Three-year vocational education

c) General secondary education

d) Undergraduate studies

e) Graduate or specialist postgraduate studies

f) Postgraduate scientific (master’s or doctoral) studies

20. Is anyone in your family a healthcare professional?

a) Yes

b) No
